# The correlation between plasma total homocysteine level and gestational diabetes mellitus in a Chinese Han population

**DOI:** 10.1038/s41598-020-75797-w

**Published:** 2020-10-29

**Authors:** Mengyao Deng, Jia Zhou, Zhao Tang, Jun Xiang, Jing Yi, Yushuang Peng, Lihua Di, Xiaobing Zhai, Mei Yang, Yukai Du

**Affiliations:** 1 Research Center for Health Promotion in Women, Youth and Children, Department of Maternal, Child and Adolescent Health, School of Medicine, Wuhan University of Science and Technology, Wuhan, 430065 China; 2 Department of Maternal and Child Health, Tongji Medical College, Huazhong University of Science and Technology, Wuhan, 430030 China

**Keywords:** Diseases, Endocrinology, Risk factors

## Abstract

To assess the correlation between plasma total homocysteine (tHcy) level and gestational diabetes mellitus (GDM) in a Chinese Han population. This case–control study included 350 GDM patients and 346 gestational week-matched normal glucose tolerance (NGT) pregnant women. Plasma tHcy and insulin levels were analyzed by HPLC and ELISA respectively. Logistic regression analysis was used to investigate the correlation between plasma tHcy level and risk of GDM. Women with GDM had a higher plasma tHcy level than NGT women (6.61 ± 1.32 vs. 6.17 ± 1.29 μmol/L, *P* = 0.001)). The GDM risk was 1.79 (*OR* = 1.79, 95% *CI* 1.18–2.72*, P* = 0.006) times higher in women whose plasma tHcy level was ≥ 7.29 μmol/L compared to women with plasma tHcy level < 5.75 μmol/L. Stratified analysis showed the GDM risk were much higher when HOMA-IR index ≥ 2 (*OR* = 5.42, 95% *CI* 2.51–11.74, *P* < 0.001), age ≥ 30 years (*OR* = 5.14, 95% *CI* 2.78–9.52, *P* < 0.001), or women with a family history of type 2 diabetes mellitus (T2DM) (*OR* = 4.13, 95% *CI* 1.78–9.56, *P* = 0.001). In the Chinese Han population, an elevated plasma tHcy level may increase the overall risk of GDM especially in women with a high HOMA-IR index, increasing age or with family history of T2DM.

## Introduction

Gestational diabetes mellitus (GDM) is a common complication of pregnancy. It is associated with a variety of maternal and infant perinatal complications, such as preeclampsia, gestational hypertension, hydramnios in mothers, and preterm birth, hypoglycemia, macrosomia in infants and so on^1^. The pathogenesis of GDM is still not fully understood, but it is very similar to that of type 2 diabetes mellitus (T2DM), which abnormal pancreatic insulin release and insulin resistance (IR) being involved^2–4^.

Homocysteine (Hcy) is a naturally occurring sulfur-containing amino acid, its potential role in various cardiovascular diseases has been investigated. In recent years, more and more studies have revealed a positive correlation between plasma tHcy level and the degree of IR. The patients with T2DM, metabolism syndrome or polycystic ovarian syndrome have a significantly higher plasma tHcy levels^5–7^. The results of in vivo and in vitro experiments also showed that hyperhomocysteinemia could promote IR by inducing endoplasmic reticulum stress or upregulating resistin production in adipose tissue^8,9^. Therefore, it is reasonable to hypothesize that elevated plasma tHcy level may be involved in the onset of GDM^10–12^. However, there are conflicting conclusions about the relationship between plasma tHcy and GDM because of the different sample size, diagnostic criteria or study population^2,10,13,14^.

Genetic background plays an important role in the pathogenesis of GDM and Hcy metabolism. Different ethnic groups present with a varying incidence of GDM, and the prevalence of GDM is higher in Asian people and is up to more than 20%^13,15^. Meanwhile, the polymorphisms of Hcy metabolic enzyme gene are quite different among different populations. Therefore, it is very important to explore the relationship between tHcy level and GDM in different populations. In China, the Han population, accounts for more than 92%, is the main population. A few studies focus on the relationship between plasma tHcy level and GDM in Chinese Han population, and their correlation is still unclear in Chinese Han population.

Based on these premises, the aim of our study is to assess the correlation between plasma tHcy level and GDM in the Chinese Han population.

## Results

### Baseline characteristics

The basic characteristics of the participants included in the study were showed in Table 1. The GDM patients were older and had a higher BMI at pre-pregnancy and enrollment, blood pressure, fasting plasma insulin (FPI), HOMA-IR than NGT pregnant women (*P* < 0.05). Meanwhile, a higher proportion of GDM patients had the family history of T2DM (23.71% vs. 10.12%, *P* < 0.001). And the plasma tHcy level was significantly higher in GDM group than NGT group (6.61 ± 1.32 vs. 6.17 ± 1.29 μmol/L, *P* = 0.001).

**Table 1:** Anthropometric and clinical characteristics of participants.

	GDM(N = 350)	NGT(N = 346)	*P* value
Age (years)	30.20 ± 1.15	27.54 ± 1.15	< 0.001
Gestational weeks	26.18 ± 1.07	26.38 ± 1.08	0.206
Pre-pregnancy BMI (kg/m^2^)	20.89 ± 1.15	19.95 ± 1.12	< 0.001
Enrolled BMI (kg/m^2^)	24.25 ± 1.13	23.43 ± 1.12	< 0.001
FPG (mmol/L)	4.68 ± 1.12	4.27 ± 1.07	< 0.001
OGTT-1 h (mmol/L)	10.00 ± 1.17	7.24 ± 1.26	< 0.001
OGTT-2 h (mmol/L)	8.91 ± 1.23	6.46 ± 1.17	< 0.001
Systolic BP (mmHg)	112.20 ± 1.12	109.65 ± 1.10	< 0.001
Diastolic BP (mmHg)	64.57 ± 1.12	63.10 ± 1.12	0.001
FPI (mU/L)	7.94 ± 1.48	6.76 ± 1.45	< 0.001
HOMA-IR	1.62 ± 1.58	1.29 ± 1.48	< 0.001
HOMA-f	163.11 ± 1.71	165.42 ± 1.69	0.780
Plasma tHcy (μmol/L)	6.61 ± 1.32	6.17 ± 1.29	0.001
**Family history of T2DM**			
Yes	83 (23.71%)	35 (10.12%)	< 0.001
No	267 (76.29%)	311 (89.88%)	

GDM, gestational diabetes mellitus; NGT, normal glucose tolerance; BMI, body mass index; FPG, fasting plasma glucose; OGTT, oral glucose tolerance test; BP, blood pressure; FPI, fasting plasma insulin; HOMA‐IR, the homeostasis model assessment for insulin resistance; HOMA‐f, the homeostasis model assessment for β‐cell function; tHcy, total homocysteine; T2DM, type 2 diabetes mellitus.

### Association between plasma tHcy levels and GDM risk

There was not significant correlation between plasma tHcy and HOMA-IR (*r* = 0.029, *P* = 0.459). The cutoff levels for the 33th, 66th percentiles of plasma tHcy levels for all participants were 5.75 and 7.29 μmol/L, respectively. And three tertiles were established based on these levels (Table 2). Multiple logistic regression analysis was carried out, using tHcy level as independent variable. And the results showed that there was a significant association between the third tertile tHcy level and GDM risk (*OR* = 1.79, 95% *CI* 1.18–2.72, *P* = 0.006), adjusted by age, pre-pregnant BMI, gravidity, HOMA-IR and family history of T2DM. Meanwhile, there were significant associations between age (*OR* = 1.17, 95% *CI* 1.12–1.23, *P* < 0.001), HOMA-IR (*OR* = 2.76, 95% *CI* 2.01–3.78, *P* < 0.001), family history of T2DM (*OR* = 3.09, 95% *CI* 1.93–4.95, *P* < 0.001) and GDM risk, respectively.

**Table 2:** Association between plasma tHcy levels and GDM risk.

Plasma tHcy levels (μmol/L)	GDM (n)	NGT (n)	Adjusted *OR* (95% *CI*)	*P*
< 5.75	95	129	Ref	
5.75–7.29	109	116	1.26 (0.84–1.90)	0.269
≥ 7.29	128	97	**1.79 (1.18–2.72)**	**0.006**

Multivariate logistic regression used the model adjusted for age, pre-pregnant BMI, gravidity, HOMA-IR and family history of T2DM.

tHcy, total homocysteine; GDM, gestational diabetes mellitus; NGT, normal glucose tolerance; *OR*, odds ratio; *CI*, confidence interval.

Statistically significant values are given in bold.

### Stratified analysis by HOMA-IR, age and family history of T2DM

As Table 3 presented, for the participants with the HOMA-IR index lower than 2, only the third tertile tHcy level concluded statistically significant relationship with GDM risk (*OR* = 1.75, 95% *CI* 1.12–2.75, *P* = 0.014). However, for participants with the HOMA-IR index higher than 2, no matter what the plasma tHcy levels were, *OR*s all were statistically significant. And when plasma tHcy higher than the third tertile, the *OR* was the highest (*OR* = 5.42, 95% *CI* 2.51–11.74, *P* < 0.001). Stratified analysis by family history of T2DM showed similar results. In addition, when participants were younger than 30 years old, there were no significant relationships between plasma tHcy level and GDM risk. But when participants were older than 30 years old, the *OR*s were significant when plasma tHcy level higher than the first tertile.

**Table 3:** The ORs of GDM by plasma tHcy levels and HOMA-IR, age or family history of diabetes.

	Plasma tHcy levels (μmol/L)	< 5.75		5.75–7.29		≥ 7.29	
		*OR* (95% *CI*)	*P*	*OR* (95% *CI*)	*P*	*OR* (95% *CI*)	*P*
HOMA-IR^a^	< 2	Ref		1.28 (0.83–1.99)	0.266	**1.75 (1.12–2.75)**	**0.014**
	≥ 2	**4.39 (1.92–10.04)**	** < 0.001**	**3.68 (1.69–8.03)**	**0.001**	**5.42 (2.51–11.74)**	** < 0.001**
Age(years)^b^	< 30	Ref		1.15 (0.69–1.90)	0.594	1.06 (0.62–1.81)	0.833
	≥ 30	1.47 (0.90–2.71)	0.217	**2.47 (1.34–4.54)**	**0.004**	**5.14 (2.78–9.52)**	** < 0.001**
Family history of T2DM^c^	No	Ref		1.10 (0.70–1.72)	0.684	**1.78 (1.13–2.79)**	**0.013**
	Yes	**2.32 (1.08–4.98)**	**0.032**	**6.31 (2.61–15.26)**	** < 0.001**	**4.13 (1.78–9.56)**	**0.001**

tHcy, total homocysteine; *OR*, odds ratio; *CI*, confidence interval; HOMA‐IR, the homeostasis model assessment for insulin resistance; T2DM, type 2 diabetes mellitus.

^a^Model adjusted for age, family history of T2DM, pre-pregnant BMI and gravidity.

^b^Model adjusted for family history of T2DM, HOMA-IR, pre-pregnancy BMI and gravidity.

^c^Model adjusted for age, HOMA-IR, pre-pregnancy BMI and gravidity.

Statistically significant values are given in bold.

## Discussion

In this study, GDM patients had a significantly higher plasma tHcy level than NGT women, and the risk of GDM was increased with elevated plasma tHcy level in Chinese Han population in both, the overall analysis and the stratified analysis. But the causal link between plasma tHcy level and GDM sitll need further studied.

During pregnancy, the women are in the state of relative IR due to the increase of insulin-like substances produced by placenta, and GDM occurs when pregnant women cannot produce enough insulin^16^. Though the pathogenesis of GDM is still unknown, it is well-established that IR plays an important role in the onset of GDM. Many studies have showed that plasma tHcy levels are positively correlated with the degree of IR^6,7^. Hence, in recent years, some studies have explored the role of plasma tHcy in GDM. But the results have been inconsistent. The serum Hcy levels in women with GDM was significantly elevated compared with normal pregnant women in the study of Guven et al.^11^. But Lai JS et al. found an association between higher Hcy level and lower odds of GDM^13^. Though the meta-analysis by our study team already indicated a higher tHcy level in GDM patients than non-GDM pregnant women^10^. The sample sizes of all studies included in this meta-analysis were small (not more than 250 for each study). And the information about the relationship between the two in Chinese Han population is still scarce.

Our results showed that in Chinese Han population, the GDM risk was increased with the increment of plasma tHcy level. But we didn’t find the correlation between the levels of plasma tHcy and HOMA-IR. We only enrolled patients without insulin or oral drug therapy. There was a possibility to underestimate the relationship between the two. In the absence of standard cutoff points for HOMA-IR score, we assumed a value of greater than 2.0 as IR^17^. And the stratified analysis showed the GDM risk was much greater when HOMA-IR index higher than 2. The relationship between plasma tHcy level and IR in the pathogenesis of GDM remains to be further explored.

Older age is accepted as an independent risk factor of GDM. As maternal age increased, the incidence of GDM increased from 2.2% in women age under 25 years to 14.7% in women over 35 years^18^. When we conducted the stratified analysis by age, we observed the GDM risk would be increased higher with increment of plasma tHcy level when the participants were older than 30 years in Chinese Han population. Some studies have reported that plasma tHcy levels increased with age^4^. But whether the different age will influence the role of plasma tHcy in the incidence of GDM is still needed to be verified.

Genetic background plays a vital role in the occurrence of GDM^4^. Family history of T2DM, was also reported as an independent risk factor of GDM in many studies^19,20^. In a meta-analysis^20^, the overall *OR* of family history of T2DM for developing GDM was estimated as 3.46 (95% *CI* 2.80–4.27), which was similar to the result in our study (*OR* = 3.09, 95% *CI* 1.93–4.95). In addition, the participants with family history of T2DM and greater plasma tHcy levels showed a significantly higher risk of GDM in our study. Animal studies have shown that prenatal exposure to diabetic intrauterine milieu leads to an increased risk in the female offspring of developing GDM^21^, and epidemiology results also showed a high prevalence of T2DM in the mothers of women with GDM^22,23^. Furthermore, in the study of Rhee SY et al. indicated that a history of T2DM in first-degree relatives (not only mother, but also father or sibling) was associated with an increased risk of developing GDM^23^. The role of plasma tHcy in GDM patients with a family history of T2DM is unclear.

Although with a larger sample size in our study, several limitations still should be noted. Firstly, the confounding factors could not be ruled out completely, such as the quantity of the dietary and exercise status. Secondly, our GDM patients were all on diet control, the effects of plasma tHcy levels on GDM risk might be underestimated. Thirdly, since this study was not prospective, causality between increment of plasma tHcy level and GDM risk could not be determined. In addition, the study was conducted among Chinese Han population, the extrapolation of the results should be considered carefully taking into account the possible differences between different populations.

In conclusion, in Chinese Han population, the pregnant women with higher plasma tHcy level might have a higher GDM risk, especially in women with a higher HOMA-IR index, older age or with family history of T2DM. In the future, the large, rigorous, perspective studies should be considered to explore the correlation between plasma tHcy level and GDM.

## Material and methods

### Study participants

386 GDM patients and 390 NGT pregnant women were enrolled in our study. After excluding women with previous GDM (n = 10), bigeminal pregnancy or polyembryony (n = 12), taking any medication (apart from iron and folate supplementation) for at least 3 months before enrollment (n = 24), known vitamin deficiency or underlying diseases, such as hypertension, cardiovascular disease, thyroid disease, pre-gestational diabetes (n = 16), and not Chinese Han population (n = 18), 350 GDM patients and 346 gestational week-matched normal glucose tolerance (NGT) individuals were included in our study finally. GDM patients were consecutively recruited between January 15 to September 31, 2015 at Maternal and Child Health Care Hospital of Bao’an District, Shenzhen, China. NGT pregnant women were randomly selected at the same outpatient during the same time. The study was approved by the institutional review boards of Tongji Medical College, Huazhong University of Science & Technology, and all participants provided informed consent for participation. The methods were carried out in accordance with the approved guidelines.

All pregnant women were routinely screened for GDM between 24 and 28 gestational weeks. A 75 g oral glucose tolerance test (OGTT) was performed. The diagnosis of GDM was based upon the criteria of International Association of Diabetes and Pregnancy Study Groups by which one or more of the following plasma values from 75 g OGTT must be equaled or exceeded: fasting 5.1 mmol/L, 1 h 10.0 mmol/L and 2 h 8.5 mmol/L^24^. Because some studies have found that the use of insulin or metformin may influence the level of Hcy^25,26^, only diet-treated GDM women were included in this study.

Both groups were enrolled during 24 and 28 weeks right after OGTT. At recruitment, blood samples (after an 8 to 12 h fast, no more than 12 h) and demographic characteristics, including age and gestational week were collected by interviewers. The physical examination included an assessment of body mass with the calculation of body mass index (BMI) at the beginning of pregnancy and on the day when the OGTT was applied.

### Laboratory testing

2 ml blood from the antecubital vein were immediately placed on ice and separated into plasma and cells within 30 min, then stored at – 80 °C until analysis. Plasma glucose measurements were performed by glucose oxidase method on the Cobas 8000 Modular Analyzer Series (Roche, Mannheim). Fasting plasma insulin (FPI) was determined by enzyme-linked immunosorbent assay (ELISA) with the Insulin ELISA 10-1113-10 kit (Mercodia, Sweden, with intraassay CV of 5.91% and between-assay CV of 7.15%). The absorbance was measured at 450 nm using the Automatic Microplate Reader (Syngene, BioTek, USA). Homeostasis model of insulin resistance (HOMA-IR) was used to evaluate insulin resistance. HOMA-IR was calculated as fasting glucose × fasting insulin/22.5. HOMA-beta cell function (HOMA-f) was calculated as 20 × fasting insulin/(fasting glucose − 3.5).

Plasma total homocysteine (tHcy: the sum of all Hcy forms) was measured by high-performance liquid chromatography (HPLC, Waters 1525, USA) with fluorescence detection, applying 7-fluorobenzo-2oxa-1,3-diazole-4-sulfonate (SBD-F, Sigma, USA) as derivating agent and tris(2-carboxyethyl)phosphine (TCEP, Sigma, USA) as reducing agent^27^. The intraassay CV was 3.60%, and between-assay CV was 4.49%.

### Statistical analysis

Normality of distribution for continuous variables was tested by the Kolmogorov–Smirnov test. Normal distribution data were described by mean ± SD and the differences between groups were compared by unpaired Student’s *t*-test. The differences of classified variables were tested using Pearson’s chi-squared test. Spearman’s bivariate correlation analyses was used to explore the correlation between plasma tHcy level and the index of HOMA-IR. Multiple logistic regression analysis was used to determine the correlation between plasma tHcy level and GDM risk. *P* < 0.05 was accepted as statistically significant. Analyses were performed by using SPSS Software, Version 25.0 for Windows (SPSS Inc., Chicago, IL, USA).
